# Gut Microbiota and Liver Interaction through Immune System Cross-Talk: A Comprehensive Review at the Time of the SARS-CoV-2 Pandemic

**DOI:** 10.3390/jcm9082488

**Published:** 2020-08-03

**Authors:** Emidio Scarpellini, Sharmila Fagoonee, Emanuele Rinninella, Carlo Rasetti, Isabella Aquila, Tiziana Larussa, Pietrantonio Ricci, Francesco Luzza, Ludovico Abenavoli

**Affiliations:** 1 Internal Medicine Unit, “Madonna del Soccorso” General Hospital, San Benedetto del, 63074 Tronto, Italy; bonimv@libero.it; 2 Department of Biomedical Sciences, KU Leuven, Gasthuisberg University Hospital, TARGID, 3000 Leuven, Belgium; 3 Institute for Biostructure and Bioimaging, National Research Council, Molecular Biotechnology Center, 10121 Turin, Italy; sharmila.fagoonee@unito.it; 4 Nephrology and Urology Department, Gastroenterology, Endocrinology, Fondazione Policlinico A, Clinical Nutrition Unit, Gemelli IRCCS, 00168 Rome, Italy; e.rinninella@gmail.com; 5 Institute of Medical Pathology, Catholic University of the Sacred Heart, 00168 Rome, Italy; 6 Institute of Legal Medicine and Department of Surgical and Medical Sciences, University “Magna Graecia” of Catanzaro (UMG), 88100 Viale Europa, Italy; isabella.aquila@hotmail.it (I.A.); ricci@unicz.it (P.R.); 7 Department of Health Sciences, University “Magna Græcia”, 88100 Catanzaro, Italy; tiziana.larussa@gmail.com (T.L.); luzza@unicz.it (F.L.)

**Keywords:** gut microbiota, gut virome, steatosis, cirrhosis, hepatocellular carcinoma

## Abstract

Background and aims: The gut microbiota is a complex ecosystem containing bacteria, viruses, fungi, yeasts and other single-celled organisms. It is involved in the development and maintenance of both innate and systemic immunity of the body. Emerging evidence has shown its role in liver diseases through the immune system cross-talk. We review herein literature data regarding the triangular interaction between gut microbiota, immune system and liver in health and disease. Methods: We conducted a search on the main medical databases for original articles, reviews, meta-analyses, randomized clinical trials and case series using the following keywords and acronyms and their associations: gut microbiota, microbiome, gut virome, immunity, gastrointestinal-associated lymphoid tissue (GALT), non-alcoholic fatty liver disease (NAFLD), non-alcoholic steato-hepatitis (NASH), alcoholic liver disease, liver cirrhosis, hepatocellular carcinoma. Results: The gut microbiota consists of microorganisms that educate our systemic immunity through GALT and non-GALT interactions. The latter maintain health but are also involved in the pathophysiology and in the outcome of several liver diseases, particularly those with metabolic, toxic or immune-mediated etiology. In this context, gut virome has an emerging role in liver diseases and needs to be further investigated, especially due to the link reported between severe acute respiratory syndrome-coronavirus-2 (SARS-CoV-2) infection and hepatic dysfunctions. Conclusions: Changes in gut microbiota composition and alterations in the immune system response are involved in the pathogenesis of metabolic and immune-mediated liver diseases.

## 1. Introduction

The human microbiota, now considered as a functional organ in se, consists of a complex community of microorganisms (bacteria, yeasts, fungi, archea, protozoa and virus), living on our skin and mucosal tissues, hence forming an efficient ecosystem with the body [1,2].

Despite the apparent alliance between gut microbiota and its host, this intimate relationship poses a permanent threat to the host’s health, requiring constant control. Thus, the role of the human immune system in fine-tuning and shaping the microbiota is of paramount importance [3].

The function of microbiota can be further extrapolated and considered beneficial or pathological beyond the gastrointestinal (GI) tract, for example in the liver. In fact, venous blood flow from the gut reaches the liver via the portal vein, carrying microbial products and inducing the host’s immunological responses to these. On the other hand, the liver produces bile that flows to the gut directly and influences the resident microbial environment [4]. This circulatory loop between liver and gut is an explicative tale of how changes in the gut flora can have both beneficial and/or harmful consequences for the host [5].

This review summarizes the evidences on the triangular interaction between gut microbiota, immune system and liver, in health and disease. Since the epidemiology of chronic liver diseases is changing, due to the decreasing rate of viral hepatitis and the increasing new epidemic of a wide spectrum of alcoholic and non-alcoholic fatty liver disease (NAFLD) [6,7], we focus our attention on non-viral hepatitis. Furthermore, due to the link reported between severe acute respiratory syndrome-coronavirus 2 (SARS-CoV-2) infection and hepatic dysfunctions, we outline the emerging role of the gut virome in liver diseases.

## 2. Methods

We conducted a PubMed and Medline search for original articles, reviews, meta-analyses and case series using the following keywords, their acronyms and their associations: gut microbiota, microbiome, gut virome, immunity, gastrointestinal associated lymphoid tissue (GALT), liver disease, non-alcoholic fatty liver disease, non-alcoholic steato-hepatitis (NASH), alcoholic liver disease, liver cirrhosis and hepatocellular carcinoma. When appropriate, preliminary evidences from abstracts belonging to main national and international gastroenterological meetings (e.g., United European Gastroenterology Week, Digestive Disease Week) were also included. The papers found from the above mentioned sources were reviewed by two of the authors (L.A. and E.S.) according to PRISMA guidelines [8]. The last MEDLINE search was performed on 30th April 2020.

## 3. Gut Microbiota, Immune System and Liver Diseases

### 3.1. Gut Microbiota Composition and Main Functions

The human GI tract hosts over 100 trillion microbes, predominantly bacteria. Intriguingly, the total number of microbes outnumbers by about ten times that of the cells of the human body [3]. Taxonomically, bacteria harbouring human gut microbiota are divided in phyla, classes, orders, families, genera, and species. A few phyla include more than 160 species [9]. The main gut microbial phyla are: *Firmicutes, Bacteroidetes, Actinobacteria, Proteobacteria, Fusobacteria*, and *Verrucomicrobia*. The two phyla *Firmicutes* and *Bacteroidetes* account for almost 90% of the entire gut microbiota with the former being composed of more than 200 different genera (e.g., *Lactobacillus, Bacillus, Clostridium, Enterococcus, Ruminicoccus* and *Clostridium*) and *Bacteroidetes* having two predominant genera (namely, *Bacteroides* and *Prevotella*) [1,9].

The collective genome of the gut microbiota (called microbiome) tends to be 150-fold bigger than that of human cells. This may explain the fact that gut microbiota composition variability inter-subjects is almost infinite [10]. Around one-tenth of the total colonizing bacterial species per individual constitute a plastic “microbial fingerprint” varying through life, starting from delivery to ageing, and subject to dietary changes and exposure to antibiotics, prebiotics and probiotics [11]. Indeed, a microbial ‘core’ intestinal microbiota includes 66 species conserved in over 50% of the general population. Nevertheless, the majority of species are individual-specific [12]. The use of culture-based methods has limited the study of gut microbiome. On the contrary, the use of new metagenomic technologies has unravelled the limitless potential for inter/intra-individual variability of gut microbiome [11].

Microbial life starts with a limited and unstable repertoire of microorganisms amenable to changes to allow evolution of a stable ecosystem. Thus, caesarean-born neonates acquire the dominant bacterial phyla, *Firmicutes* and *Bacteroidetes*, at a later stage than those born transvaginally. On the other hand, infants born transvaginally have a more precocious skin and oral microbiota colonization [13].

The first year of neonatal life frames a critical window, shaping the composition of the microbiota, influenced primarily by maternal-neonate interactions [14]. Changes in gut microbiota ensue through adolescence until a stable asset is reached in adulthood. This setup is variably modulated by diet, lifestyle, drugs/substances/food use and abuse until another shift in the elderly and very ultra-elderly occurs [15].

Gut microbiota is crucial for nutrients absorption and fermentation, regulation of intestinal permeability (IP), host metabolism (e.g., carbohydrates absorption and processing, proteins putrefaction, bile acids formation, insulin sensitivity) and last but not least, modulation of intestinal and systemic immunity, thus maintaining antigen tolerance and avoiding pathogen expansion [16].Thousands of years of microbial and immune bidirectional evolution have created a harmonious co-existence that can be disrupted and re-established in a continuous manner both in health and disease in humans [3,17].

### 3.2. GALT and Non-GALT Systems and Their Interactions with Gut Microbiota

**Figure 1:**
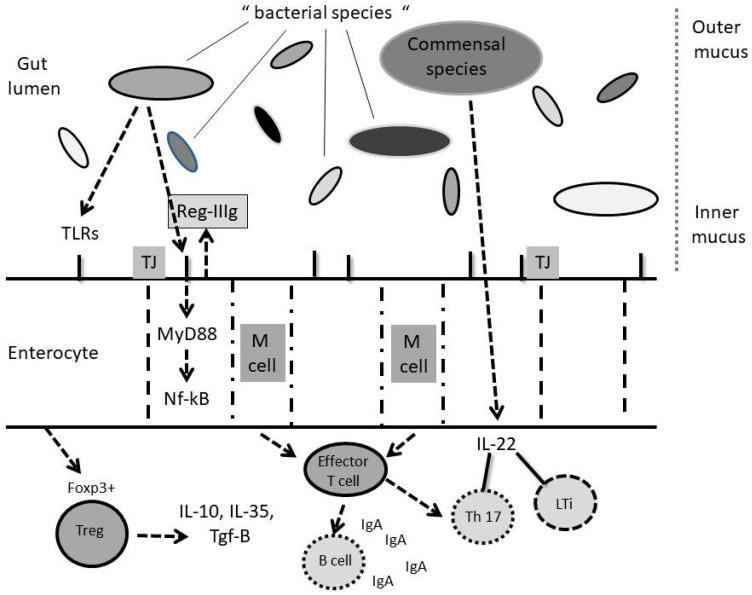
Example of microbial-immune interplay through intestine in hepatic diseases. M cells “sense” gut microbiota and educate mucosal immunity. In particular; Toll Like receptors (TLRs) on the enterocytes’ surface sense microbe associated molecular patterns (MAMPs) and pathogen associated molecular patterns (PAMPs) are allowed to pass through tight juctions (TJ) among cells with production of nuclear factor (NF)k-B via the MY-D88 pathway. This results in anti-microbial peptides production (namely, RegIIIy) that regulate the resident gut microbiota.

The small intestine itself is a barrier towards the environment. In fact, it consists of one mucosal layer with epithelial cell-derived antimicrobial peptides (RegIIIγ) that prevent bacterial penetration through the mucus layer [18,19] (Figure 1).

Gut microbiota composition changes throughout the entire GI tract. This variation depends on different environmental conditions of the diverse tracts. More specifically, one of these environmental conditions is represented by changes in IP, resembled by alterations in the tight junctions (TJ). TJ are plastic gates for the translocation of microbial antigens and drive systemic inflammation. In fact, changes in the expression of claudin (one of the proteins constituting the TJ) have been associated with the development of colitis in animal models [20,21]. On the other hand, tight junctions closing is impaired by various inflammatory cytokines [22]. There is also a putative role for modified claudin expression in mucosal immunity dysfunctions [22]. More recently, it has been shown that activation of myosin light chain kinase (MLCK), by the cytokines tumor necrosis factor (TNF) and interferon (IFN)-γ, may affect mucosal permeability through the endocytosis of occludin proteins belonging to TJ [23]. Furthermore, MLCK can also be activated by *Escherichia coli* (*E. coli)* bacterial lipopolysaccharide (LPS) and interleukin (IL)-1β [22].

The role and behaviour of gut microbiota in the modulation of GALT has been clarified by experiments on germ-free animals [24]. GALT is composed by Peyer’s patches and mesenteric lymph nodes [25]. Although GALT tolerance is genetically programmed, its maturation and development (e.g., isolated lymphoid follicles—ILFs) are dependent on the environment [26]. Indeed, germ-free mice have hypoplastic Peyer’s patches/mesenteric lymph nodes but no ILFs in the small intestine [27]. Prenatal Peyer’s patches and mesenteric lymph nodes functioning is driven by pro-inflammatory lymphoid tissue inducers (LTi), innate lymphoid cells able to recruit and send B and T lymphocytes into B-cell follicles and T-cell zones, respectively, in the absence of microbiota [27]. Postnatally, ILFs are also driven by LTi cells but only after microbiota colonization of the GI tract. Therefore, ILFs are able to control gut homeostasis through microbes. In fact, mice with LTi cells dysfunction have an overgrowth of anaerobic, Gram-negative bacteria in the gut [28].

GALT is able to inform and educate both the innate and adaptive immune system through antigen-sampling of gut microbiota via specialized M cells [28,29]. Microbe-associated molecular patterns (MAMPs) (e.g., peptidoglycan, LPS) can be recognized by several pattern recognition receptors present on enterocytes’ surface (namely, toll-like receptor (TLR) and cytosolic nucleotide-binding oligomerization domain (NOD)-like receptor), resulting in ILFs development and production of other antibacterial proteins [29]. On the other hand, gut microbiota is also able to modulate signal transduction through interaction with enterocytes. This process helps in maintaining a microbial balance, hence preserving host health [3].

### 3.3. The Immune System

#### 3.3.1. Innate Immunity

TLRs activate downstream signals primarily facilitated by the adaptor protein MyD88. This process seems to be crucial for survival as indicated by MyD88 deficient control animals [30]. This step helps immune system to recognize commensal from pathogenic bacteria [3].

When commensal bacteria are recognized by TLRs, they induce a significant production of cytoprotective cytokines, heat-shock and anti-microbial proteins. In fact, Biswas et al. showed that TLR signalling downregulation by protein IRAK-M is able to protect from colitis development by maintaining intestinal microbiota homeostasis [31].

Moreover, innate NOD-like receptors (NLRs) help in the maintenance of gut microbial homeostasis. Similar to TLRs, these are intracellular proteins able to activate nuclear factor (NF)-κB and other transcriptional factors, the mutations of which are implicated in the pathogenesis of inflammatory bowel diseases (IBD) [32,33,34]. Importantly, a subset of NLRs can activate caspase-1 through the assembly of the inflammasome, a multiprotein complex associated with the production of interleukin IL-1β and IL-1, which are protective against colitis development [35].

#### 3.3.2. Adaptive Immunity

Adaptive immunity involves both T and B cells. T cells’ highly diverse receptors are able to recognize distinct molecular sequences; B cells have other receptors generated by somatic hypermutations. Altogether, these receptors allow a highly specific, direct immune response and generate the well-known immunological memory that is the core of adaptive immunity [36].

T and B cells interact via a continuous crosstalk (Figure 1). Gut microbiota educate and stimulate T lymphocyte subsets in the intestinal lamina propria. This has been shown in germ-free animals with T cell deficiencies that are partially restored by gut microbiota reshuffling [37]. These features are typical of immune-mediated allergies and hypersensitivities [38].

Gut colonization with single filamentous bacteria can lead to the induction of IL-17 and IL-22 secreting CD4+ lymphocytes (Th17 cells) [39], formerly associated with *Helicobacter pylori*-induced gastritis [40]. However, the induction of these effector T cells is crucial in lowering the number of pathogenic bacteria. Indeed, mice lacking single filamentous bacteria colonization cannot counteract the growth of the pathogenic *Citrobacter rodentium*, a strain very similar to the human-associated pathogen *E. coli* [39,40]. Not only the single filamentous bacteria but also typical dendritic resident intestinal CD70^high^CD11c^low^ antigen-presenting cells interacting with adenosine triphosphate (ATP) are able to regulate Th17 differentiation [41].

Finally, the quick shift towards a pathogenetic immunological environment may affect gut microbiota. For example, non-pathogenic *E. coli* and *Enterococcus faecalis* (*E. faecalis*) are capable of inducing an aggressive Th1/Th17 pancolitis in IL-10 knockout mice, thus further altering the gut microbiota [41].

Regulatory T cells (namely, T_regs_) can suppress the intestinal inflammation and maintain commensal microbiota tolerance through a mutual interaction [42]. These cells represent 1–3% of circulating CD4+T cells and have a high expression of CD25 as well as of intracellular transcription factor forkhead box P3 (FOXP3) [43,44]. Notably, their concentration is higher in the gut [45,46]. They inhibit the effector T lymphocytes (Th1, Th2 and Th17) and antigen-presenting cells [46,47], mainly through the production of IL-10 [47]. Indeed, germ-free mice show reduced levels of T_regs_ in the colon [48].

From an evolutionistic point of view, it is conceivable that gut microbiota has evolved enhancing these natural anti-inflammatory T cells components (namely T_regs_). DNA sequencing has unravelled the microbial-immune system interactions. For instance, polysaccharide A, a bacterial component of the commensal bug *Bacteroides fragilis*, is able to promote the differentiation of IL-10-producing T_regs_ through an interaction with TLR2 expressed on T cells [49,50]. In addition, Gram-positive *Clostridia* colonization prevents the development of dextran sulphate sodium (DSS)-induced colitis through a T-reg-mediated mechanism [50,51]. Furthermore, fermentation of complex carbohydrates by the microbiota leads to the production of short chain fatty acids (namely, acetic acid, propionic acid and butyric acid) in the colon. These products also induce T_reg_ proliferation [51]. On the contrary, the recently recognized microbial TLR ligand, cytosine–guanine (CpG)-containing DNA, can have both direct and indirect suppressive effects on T_regs_ [52].

A recent paper by Wesemann et al. has shown that the very first B cells can develop in the intestinal mucosa with the production of modulating immunoglobulins (Ig) [53]. Germ-free mice colonization with commensals gut bacteria are able to increase recombination activating gene (RAG) endonucleases, involved in the production of both heavy and light Ig chains [53]. This microbial-dependent maturation of B cells is crucial for removing autoreactive B cells responsible for autoimmunity. In fact, in systemic lupus erythematosus, B cells are deficient in gut homing receptors [54,55].

All the evidences considered so far support the ability of the gut microbiota in educating our GALT- and non-GALT-associated immune systems. Within this chain of events, gut microbiota helps GALT to produce B cells. In particular, the production of IgA involves CD40-CD40L interactions among B and activated T cells. Thus, the strict and complex interplay between B cells and gut microbiota supports the hypothesis that microbial diversity is responsible for regulatory B cells formation [56].

More in particular; commensal (e.g., single filamentous bacteria) and/or probiotic strains are able to induce the development of T helper 17 cells (Th17); regulatory T cells (Tregs) can produce immunoregulatory cytokines (e.g., IL-10 TGF-β and IL-35) balancing the mutual coexistence of the microbial species. Th17 cells and lymphoid tissue inducers (LTi) through IL-22 production, and the consequent step-down in RegIIIy production, further reshape the gut microbiota. Finally, B cells produce secretory IgA (sIgA) following CD40-CD40L T cell interactions with another immuno-mediated balancing effect on gut microbiota.

### 3.4. Gut Microbiota Derangements in Liver Diseases through Immune System Alterations

The gut microbiota has a clear role in the physiopathology of liver diseases. Small quantities of intestinal bacterial antigens can, through increased IP, enter the portal venous blood flow and trigger GALT- and non-GALT-based immune responses. Bacterial translocation is harmful for NAFLD pathogenesis, hepatic encephalopathy and spontaneous bacterial peritonitis development in liver cirrhosis patients [57]. The liver, however, can maintain a sensitive balance between protective immune response against exogenous antigens and immune tolerance through the large number of immune cells belonging to both innate and adaptive immune systems [58,59,60].

The strict association between gut microbiota imbalance or dysbiosis and hepatic encephalopathy was first reported in humans in the 1950s by Phillips et al. They found that nitrogenous-compounds, such as ammonia, produced by microbial-ingested proteins putrefaction, could escape hepatic detoxification, resulting in accumulation of these across the blood-brain barrier until coma develops [61].

#### 3.4.1. Alcoholic Liver Disease

Although the hepato-toxicity of alcohol is well-known, its disruptive effects cannot be attributed to toxicity only. Increased bacterial endotoxin and DNA levels are found in the systemic circulation of alcoholic liver disease patients. Bacterial LPS can activate both systemic and resident immune cells through TLR4 signalling with the induction of pro-inflammatory cytokines, forming a positive feedback loop [62]. Bacterial DNA is recognised by TLR9 that triggers the liver LPS-related inflammatory cascade [63].

However, alcoholic liver disease natural history also regards another pathophysiological mechanism involving gut microbiota. Chronic excessive alcohol consumption can lead to a significant increase in the total number of Gram-negative anaerobic bacteria of faecal origin within the jejunum [64]. Another study reported that mice chronically exposed to alcohol showed increased presence of species belonging to the *Bacteroides versus Firmicutes* phyla [65]. Finally, chronic excess alcohol intake can also lead to deregulated intestinal mycobiosis (with reduced fungi diversity and richness) and hepatic inflammation in mice [66]. In humans, marked intestinal fungal dysbiosis was also observed in alcohol-dependent patients with a significant difference among alcoholic liver disease, alcoholic steatohepatitis and liver cirrhosis [67] (Table 1).

Chronic alcohol consumption also impairs barrier immunity as ethanol inhibits natural killer cell responses with contemporary depletion of other types of lymphoid cells. Therefore, alcohol-related dysbiosis increases the susceptibility to infections which is a very severe complication in alcoholic liver disease patients with liver cirrhosis [69].

#### 3.4.2. Non-Alcoholic Fatty Liver Disease

The rapid and even more consistent epidemic of obesity in the Westernized societies, has occurred during the last 40 years and has recalled our attention on its terrible implications for health in terms of morbidity and mortality [70]. NAFLD includes a spectrum of hepatic manifestations ranging from steatosis to liver cirrhosis and, sometimes, leading directly from NASH to hepatocellular carcinoma development [71]. NAFLD is a peculiar condition associated with obesity, type 2 diabetes and insulin resistance, in the absence of significant alcohol consumption. Its histopathology is somehow indistinguishable from the alcoholic steato-hepatitis [72]. More unexpectedly, NALFD pathogenesis is similar to those of alcoholic liver disease. LPS triggering of systemic micro-inflammation is the hallmark of the triangular relationship between obesity, insulin resistance and liver steatosis/hepatitis [73]. Another peculiarity of this physiopathology is represented by the bi-directional changes occurring in obesity and gut microbiota [73]. In fact, obesity itself, with or without a high fat-diet intake, can shape the gut microbiota. On the other hand, this “obese” microbiota can reprogram the gut as well as the entire body to maximize nutrient absorption and an accumulative metabolism [74]. Furthermore, this shift in microbial populations has been associated with a metabolic endotoxaemia due to higher LPS passage through an impaired IP [75]. Who is responsible for this altered IP remains an open question in NAFLD physiopathology. An altered “dysmetabolic” gut microbiota could be the answer [76,77,78]. This obesogenic intestinal microbiota has been linked to the development of insulin resistance through the LPS/TLR4/CD14 systems [79]. Once again, the immune response is crucial to close the physiopathologic ring between diet, microbiota and diabetes/insulin resistance [80] (Table 1).

#### 3.4.3. Autoimmune Diseases

Autoimmune hepatic diseases include several pathological entities, named autoimmune hepatitis (AIH), primary biliary cholangitis (PBC) and primary sclerosing cholangitis (PSC), characterized by antibody formation to self-antigens. These diseases do not have a selective hepatic manifestation but are systemic with phenotypic diversity and grading [81].

Recently, convincing data on the association between the influence of gut microbiota and the diffusion of these diseases have been presented. In fact, almost 20% of chronic hepatitis in the Caucasian population have hypergammaglobulinaemia and liver-directed autoantibodies. The consequent histopathological feature is the hepatic lymphocytic infiltration and subsequent hepatocellular injury as revealed in murine liver where significant TLR4 signalling correlates with the consequent trapping of CD8+ T cells [82]. TLR9 was also shown to mediate the process of homing and activation of hepatic natural killer (NK) T cells via the hepatic immune guardians, namely Kupffer cells [34]. IL-10 is another cytokine crucial for autoimmune hepatic damage regulation according to data on animals. In fact, lack of IL-10 abolishes the induction of T_regs_ and the consequent suppression of autoimmune colitis in mice via TLR4 expression on intestinal CD4+ T cells [83]. These data suggest that gut-derived products such as pathogen associated molecular patterns (PAMPs) are able to regulate T cell function within the liver.

PBC is an immune-mediated liver disease caused by immune cell activation with direct damage of intrahepatic bile ducts; almost 95% of these patients present with anti-mitochondrial antibodies at the biochemical check [84]. Hopf et al. showed a significant association between *E. coli* rough form and the presence of lipid A, a lipid component of the endotoxin responsible for germ’s toxicity, within the liver of PBC patients but not in healthy subjects. This association seems to be disease-specific [85]. Thus, pharmacological modulation of *E. coli* subpopulations might be a treatment option in PBC patients.

PSC is a progressive autoimmune disease, characterized by the complete destruction of intrahepatic and extrahepatic bile ducts, inhibition of bile acid secretion and chronic hepatocellular injury until liver cirrhosis develops [68]. In PSC patients, the pathophysiological link between gut microbiota, bowel and liver is more evident. Indeed, almost 75% of PSC patients show signs of IBD, mainly ulcerative colitis (UC). In addition, experimental models of IBD bearing pathogenic gut microflora have shown hepatic periportal inflammation [86]. These reports confirmed that intestinal microbial factors may initiate the immune response which leads to liver damage, even in the absence of underlying immune cell disease (Table 1).

#### 3.4.4. Liver Cirrhosis

In patients with liver cirrhosis, the advanced stage of chronic hepatitis that may evolve to hepatocellular carcinoma, an altered gut microbiota might play an important role [87,88] under several aspects.

Delayed bowel motility, reported during cirrhosis and potentially responsible for small bowel bacterial overgrowth, increases the time of contact of faeces with the enterocytes. Moreover, altered IP allows bacterial translocation to the systemic venous blood circulation and finally to the liver [89,90].

Recently, impaired bile secretion has been shown to be another source of bacterial translocation. In liver cirrhosis, the level of bile salts is significantly reduced, thus lowering the stability of the gut microbiota. The load of bacteria belonging to the *Clostridiales* order and responsible for the metabolism of bile salts, was found significantly reduced [91] while a higher number of the potentially pathogenic *Enterobacteriaceae* were detected in liver cirrhosis patients *versus* controls [92].

Furthermore, urease-producing bacteria (e.g., *Klebsiella* and *Proteus species*) have been associated with increased production of ammonia and LPS, both involved in the pathogenesis of hepatic encephalopathy and spontaneous bacterial peritonitis [93,94].

Finally, Qin et al. reported a difference of 75,245 microbial genes between liver cirrhosis patients and healthy subjects using the newest quantitative metagenomic methods; about 50% of the bacterial species were of buccal origin thus justifying the hypothesis that oral bacteria could invade the gut of liver cirrhosis patients [95] (Table 1).

Hepatocellular carcinoma is a common complication of liver cirrhosis and, in some contexts (as NASH), also of non-liver cirrhosis conditions. The pathogenesis of this malignancy involves chronic liver inflammation, with continuous cell death and regeneration processes [96]. Genetic TLR4 inactivation, gut microbial deprivation or germ-free status decrease the development of hepatocellular carcinoma hepatocellular carcinoma in almost 80% of cases [97]. However, pathogenic changes in the immune system have been implicated in hepatocellular carcinoma development. These include leucocyte dysfunction with reduced phagocytic activity of reticulo-endothelial cells (that is, Kupffer cells) [98,99,100], reduced antibody- and complement-mediated bacterial killing [101] and reduced proliferation of intraepithelial lymphocytes [102]. Altogether, these mechanisms explain the dysbiosis occurring in the cirrhotic patients that, in turn, enhances hepatocellular carcinoma progression (Table 1).

Despite substantial improvements in short-term outcome, liver cirrhosis, in the very last stages, continues to have a poor prognosis [103]. Thus, liver transplantation remains the only treatment option for end-stage liver disease [103]. Immunosuppression and an altered entero-hepatic bile recirculation due to anatomical changes, however, after transplant may both play a significant role in reshuffling intestinal microbial populations. In fact, in cynologous monkeys, the immunosuppressant alemtuzumab induced a complete alteration of gut microbiota with reduction of predominant Bacteroides species and increase of Enterobacteriaceae [104].

In humans, fecal microbial diversity assessment in both the pre- and post-transplant period, by immune profiling, revealed poor microbial diversity, with reduction in several commensal species and increase in pathogenic ones, such as *Enterobacteriaceae* and *Enterococcus* species. Surprisingly, this dysbiosis resolved overtime after transplantation, especially when bacterial prophylaxis was stopped and immunosuppressive regimens were reduced [105] (Table 1).

### 3.5. Gut Virome and Liver Diseases at the Time of SARS-COV-2 Pandemic

The existence of a gut virome has been very recently recognised despite the fact that pathogens (such as Norwalk virus, Rotavirus and Enterovirus) have been long-known to be found in the human intestine [106,107,108]. With advances in metagenomic technologies, novel enteric eukaryotic viruses such as *Adenoviridae, Picornaviridae, Reoviridae* families, were found to be responsible for acute diarrhoea in children’s small bowel enteropathy in developing areas of Australia [109,110]. Giant DNA viruses that infect human intestinal parasites (namely, amoebae) are mainly represented by *Mimiviridae, Mamaviridae, Marseilleviridae*. Mimiviruses have been sometimes associated with pneumonitis and diarrhoea in humans [111]. Plant-derived viruses are also present in human faeces. They are represented by pepper mild mottle virus (PMMV), oat blue dwarf virus, grapevine asteroid mosaic associated virus, maize chlorotic mottle virus, oat chlorotic stunt virus, panicum mosaic virus, and tobacco mosaic virus [112].

Intestinal bacteriophages account for around 90% of the entire gut virome [106,107]. They are commonly described as viruses of bacteria or bacterial parasites due to the ability to inject their genome into their host, integrating with its genetic material (prophage state) and inducing other phage particle synthesis resulting in bacterial cell lysis (lytic state) [106,113]. Bacteriophages have double-stranded DNA (dsDNA) [113], although single-stranded DNA (ssDNA) types are found amongst the *Microviridae* family [112]. *Microviridae* are small icosahedral viruses with circular ssDNA genomes and their members are divided into microviruses (genus *Microvirus*), gokushoviruses (subfamily *Gokushovirinae*) and *Alpavirinae* [114].

The human gut virome maintains stability and generates diversity of the human gut microbiome in dynamic equilibrium with the host via immune system tolerance [114]. Gut virome genes are also implicated in human metabolism, inflammation and carcinogenesis modulation [114]. Recent evidence points out to a new role of bacteriophages in liver metabolism and immune response regulation in humans [115].

As previously mentioned, gut microbiota promotes ethanol- induced liver disease in mice but little is known about the specific microbial factors that are responsible for this process. The presence of *E. faecalis* correlates with the severity of liver disease and with mortality in patients with alcoholic liver disease. Duan et al. recently showed that bacteriophages were able to decrease cytolysin expression in the liver and abolished ethanol-induced liver disease in humanized mice [116]. Cytolysin is a bacterial exotoxin (or bacteriocin) that is produced by *E. faecalis* but also by eukaryotic cells [117,118]. Alcoholic liver disease can be transmitted via faecal microbiota. Duan et al. found no multi-collinearity between the detection of faecal cytolysin-encoding genes and other cofactors in mice. This indicates that cytolysin may be considered an independent predictor of mortality for alcoholic liver disease. Moreover, cytolysin production is a transportable trait among *E. faecalis* isolates. Indeed, it includes both chromosomally encoded pathogenicity islands and plasmids [119]. These results confirm that the presence of cytolysin-producing *E. faecalis*, rather than the total number of bacteria, determines the severity of alcoholic liver disease and associated mortality.

Ethanol-induced changes in the gut barrier are necessary for the translocation of cytolytic *E. faecalis* from the intestine to the liver, suggesting that this bacterium may promote ethanol-induced liver disease after abnormalities of IP, as reported in mice [116]. Cytolysin-induced hepatocyte cell death may be mediated by pore formation resulting in cell lysis, independently of ethanol [116,119].

*E. faecalis* bacteriophages are highly strain-specific, can be easily isolated and, in the perspective of future therapeutic implications, present a potential for direct editing of gut microbiota [120]. Duan et al. isolated four distinct phages from sewage water. These phages can lyse the cytolytic *E. faecalis* strain isolated from Atp4aSl/Sl mice. All four phages were podophages of the virulent Picovirinae group. Importantly, administration of *E. faecalis* phages significantly reduced levels of hepatic cytolysin and faecal concentration of *Enterococcus.* Furthermore, phages administration (with siphophage or myophage morphology) did not affect the overall composition of the faecal microbiome, intestinal absorption or hepatic metabolism of ethanol [116].

In mice, the phages against cytolytic *E. faecalis* abolished ethanol-induced liver injury and steatosis, lowering the levels of transaminases (ALT), the percentages of hepatic cells positive for terminal deoxynucleotide transferase-mediated dUTP nick-end labelling, and reducing the levels of hepatic triglycerides and oil red O-staining, compared to control phages (namely, against *C. crescentus*) [116]. It can be hypothesized that treatment with lytic phages can attenuate the ethanol-induced liver disease also in humans. However, since phages can induce a strong immune reaction, safety studies are required [121]. Overall, these data are promising and suggest that cytolysin may be used as a predictive biomarker of alcoholic liver disease shifting to alcoholic steatohepatitis.

In AIH, patients are typically treated with steroids and show a good response profile. In cases where immunosuppressive therapy does not offer any benefit, and the side effects are seriousleading to the development of malignancies, bacteriophage-based approaches have been considered. Bacteriophages have been increasingly recognized as immunomodulators contributing to immune homeostasis and curbing inflammation [122]. Phages have been shown to down-regulate the expression and/or production and activity of factors associated with hepatic injury (e.g., reactive oxygen species, TLR-4 and NF-kB activation, pro-inflammatory and pro-coagulant activities of platelets) and up-regulate the expression and/or production of protective factors (e.g., IL-10, IL-1 receptor antagonist) [121]. Phages may modulate the immune response, contributing to maintenance of immune homeostasis in the GI tract and, possibly, in other sites [123,124]. Furthermore, they can diminish T cell activation, alloantigen-induced Ig production in vitro and extend the skin allograft survival in naive and sensitized mice [123,124]. In addition, phages may reduce autoimmune reaction in a mouse model of autoimmunity (namely collagen-induced arthritis) [125]. Skin and organ inflammatory infiltration induced by alloantigens and endotoxin can also be reduced markedly by phage or a phage protein administration [126]. More importantly, phages do not impair the ability of granulocytes and monocytes to kill bacteria. Clinical phage therapy decreased inflammatory markers (e.g., C-reactive protein [CRP], erythrocyte sedimentation rate, leukocytosis), even though eradication of pathogens was not achieved [127].

Liver macrophages or Kupffer cells are of paramount importance for maintenance of liver and immune systemic homeostasis [128]. In fact, deletion of Kupffer cells in experimentally-induced hepatitis suppresses liver damage and, also, collagen-induced autoimmune arthritis in mice [129,130]. Kupffer cells may modulate liver allograft tolerance implicating that transplanted subject survival may be accomplished without concurrent immunosuppression [131]. Moreover, when liver and kidney are transplanted simultaneously the liver becomes immunoprotective for the kidney [132].

Targeting pathogenic Kupffer cells may be a novel promising approach in acute and chronic liver diseases. From seventy to ninety per cent of phages administered intravenously in mice are taken up by liver [133]. Liver Kupffer cells are primarily responsible for this uptake and are unable to prime lymphocytes for antibody responses against phages. In contrast, almost the entire humoral response to phages is attributable to spleen [134].

If enhanced phagocytosis by Kupffer cells may translate into attenuation of autoimmune-mediated hepatitis, it may be expected that phage uptake by Kuppfer cells may also mediate similar effects [133]. Specifically, phage-induced decrease of reactive oxygen species and enhancement of IL-10 production by these cells may also contribute significantly to achieving immune homeostasis.

Phages induce IL-10 production by human mononuclear cells [135]. This cytokine, known for its anti-inflammatory action, plays a protective role against hepatic injury. It also has anti-fibrotic properties [136]. IL-10-producing T cells prevent liver damage in chronic hepatitis C virus infection [137]. Phages can have a moderate inhibitory effect on the activation of NF-kB, thus inhibiting liver inflammation and injury [138].

Biliary epithelial cells express TLR-4. There is increasing evidence that this receptor plays a key role in HCV infection and replication. TLR-4 has been identified as a factor associated with a high risk of developing cirrhosis in patients with chronic hepatitis C. Moreover, TLR-4 activation has been associated with the progression of other chronic liver diseases, such as AIH, PBC and PSC. Inhibitors of TLR-4 are being tested in the hope that they might prevent the progression of chronic hepatitis [139,140]. In addition, purified phages may down-regulate TLR-4, leading to lower hepatic injury with subsequent lowered hepato-carcinogenesis [141]. Of note, antiplatelet therapy prevents the development of hepatocellular carcinoma. Phages may also be part of this process, as they inhibit platelet adhesion to fibrinogen [142]. Finally, phages could also be used for the development of vaccine against hepatitis B virus and production of nanomolecules displaying peptides that could interfere with attachment of pathogenic viruses and their entry into liver cells [143].

To date, SARS-CoV-2 is responsible for a tremendous pandemic that has changed clinical as well as social behaviours. The relative collection of clinical manifestations, namely COVID-19, includes not only pulmonary abnormalities but is a systemic disease, involving the heart, liver, pancreas and kidneys. SARS-CoV-2 also affects circulating lymphocytes and the immune system [144,145,146]. Liver damage can occur during disease progression and/or as consequence of COVID-19 treatment in patients with or without pre-existing liver diseases [146]. Overall, the incidence of elevated serum transaminases in hospitalized COVID-19 patients, and, less frequently, bilirubin, ranges from 14% to 53% [147]. Moreover, liver derangement is observed more commonly in male patients and in those with more severe disease [148].

Hitherto, there is no evidence of acute or acute on chronic liver failure in COVID-19 patients [147,148]. Retrospective studies, with large cohorts, have shown that a small percentage had pre-existing hepatitis B [148]. Histopathologically, the liver of COVID-19-affected patients shows moderate microvascular steatosis and mild lobular and portal activity, indicating that the injury could have been caused by either SARS-CoV-2 infection or drugs [149]. Due to the novelty of COVID-19, we can only report putative mechanisms leading to liver damage: immune-mediated injury due to the dramatic inflammatory storm following the first week of SARS-CoV-2 infection [150]; direct cytotoxic damage due to viral replication within hepatic cells through ACE-2 receptor binding [151]; viral-induced endothelial injury and/or microthrombotic events; anoxia due to respiratory failure; drug-induced liver injury (DILI) (e.g., due to use of lopinavir/ritonavir, remdesivir, chloroquine, tocilizumab, uminefovir, Chinese traditional medicine which are potentially hepatotoxic in some patients) [146,151]. It is also noteworthy that drugs like tocilizumab and baricitinib can cause HBV reactivation, thus leading to liver failure.

It is not yet clear whether COVID-19 impairs cholestasis in patients with pre-existing cholestatic liver diseases [152]. However, the outcome of patients with liver injury is generally favourable as alterations of liver transaminases are transient and often without fatal exitus. Thus, COVID-19 liver features and preliminary evidence reported in literature raise open issues: disease evolution history will provide details about the exact pathogenesis of liver manifestations following COVID-19; the putative role for biliary tract cells in shedding the infection to the intestinal cells (also expressing ACE2); the real incidence of DILI during the treatment of COVID-19; the eventual susceptibility of patients with pre-existing liver disease to COVID-19 disease (e.g., the possible protective role of immunosuppressant *versus* disease severity); the prognostic weight of pre-existing liver disease on COVID-19 survival.

### 3.6. An Example of Gut Microbiota Modulation through Immune Interaction in Liver Disease: The Case of Probiotics

Evidence on the efficacy of gut microbiota modulation in liver cirrhosis natural history comes from studies on the use of prebiotics. Prebiotics, usually plant fibres and other non-digestible fermentable carbohydrates that lead to preferential intestinal microbial growth, have been first used in liver cirrhosis patients [153]. Lactulose is able to reverse and improve hepatic encephalopathy and the add-on positive effect on the usage of rifaximin, an antibiotic poorly absorbed at the intestinal level, support its enormous therapeutic potential in altering intestinal microbial communities to revert disease progression [3]. Moreover, lactulose, as a non-absorbable disaccharide, lowers colonic pH, improves excretion of ammonia, stimulates growth of *Bifidobacterium* and *Lactobacillus* [154].

Probiotics are defined as “live microorganisms beneficially affecting human health” [155]. Symbiotics are a combination of the prebiotics and probiotics [156]. alcoholic liver disease has been linked to an over-population of Gram-negative microbial species in the gut [157]. Studies on animal models showed the potential of *Lactobacillus GG* in reducing the severity of alcoholic hepatitis. The latter is linked to the complex mechanism of action of this probiotic that causes a reduction in gut leakiness, oxidative stress and liver inflammation [158].

In human studies, the add-on use of other probiotics, namely *Bifidobacterium bifidum* and *Lactobacillus plantarum*, was able to reverse the intestinal microbial dysbiosis with a simultaneous improvement in alcoholic liver disease features [159]. From an immunological point of view, Stadlbauer et al. showed the immune-modulator effect of *Lactobacillus casei Shirota* that was able to restore neutrophils’ phagocytic capacity, inversely correlated with an increased risk of mortality in alcoholic liver disease patients [160].

NAFLD and NASH are the most studied models in which gut microbiota and immune system dysfunction are strictly linked in determining liver damages until liver cirrhosis and hepatocellular carcinoma development [161]. In fact, data from animal studies have provided indications on the efficacy of prebiotics, probiotics and symbiotics in NAFLD treatment. Li et al. showed that 4 weeks of treatment with VSL#3, containing *lactobacillus*, *bifidobacterium* species and a streptococcal strain, was associated with improved NAFLD histology, with a reduction in hepatic total fatty acid content, and reduced serum aminotransferases levels in *ob*/*ob* mice fed with a high fat diet. These effects paralleled a significant reduction in Jun-Kinase (JNK) activity and DNA-binding activity of NF-kB [162]. In humans, a study by Loguercio et al. confirmed the capability of VSL#3 to reduce these parameters, especially with a significant decrease in lipid peroxidation, in NAFLD patients [163]. In addition, recent data supported the efficacy of gut microbiota modulation in changing not only the GALT-associated immunity but also the systemic inflammatory response. Reduced levels of LPS were found after probiotic administration in patients with NAFLD [164,165]. Malaguarnera et al. also showed that probiotics and fructooligosaccharides administration was superior to lifestyle changes in NAFLD subjects in reducing inflammatory marker levels. Levels of TNFα, endotoxin and the NASH activity index were significantly reduced by probiotics add-on use [166]. However, evidence supporting a curative role for probiotics in NAFLD, NASH and its subsiding systemic micro-inflammation process has not yet been confirmed by larger population-based studies [167].

Within the array of biliary tract liver diseases, PSC is one of the most studied autoimmune liver diseases in terms of gut microbiota modulation. In a pilot study by Vleggaar et al., patients with PSC and IBD received a multi-strain probiotic for three months without benefits in terms of symptoms relief or improvement in both liver function indexes and bile salt levels [168,169,170].

As reported above, gut microbiota and its interaction with immune system have been implicated in the pathophysiology of major complications of liver cirrhosis. Thus, research focused on microbial re-modulation, in order to reverse liver cirrhosis natural course [171]. A symbiotic preparation was used by Liu et al., who reported a significant improvement in Child-Pugh class (that is associated with prognosis) staging in about half of the patients treated, accompanied by reduction in the levels of circulating endotoxin [172].

Probiotics may have a potential as add-on treatments to prevent spontaneous bacterial peritonitis occurrence, to promote the growth of protective anaerobic organisms, but also to reduce IP [172] and GALT activation [59]. However, neither preliminary animal studies [173] nor clinical data support the efficacy of probiotics add-on to antibiotics in preventing primary or secondary spontaneous bacterial peritonitis [174]. On the contrary, the potential efficacy of probiotics in hepatic encephalopathy treatment is supported by the evidence of the beneficial effect on colonic non-urease producing bacteria that can reduce the total amount of ammonia reaching the portal system [175]. Thus, high oral doses of *Lactobacillus acidophilus* have been shown to be beneficial in improving hepatic encephalopathy [176,177]. These findings were confirmed in patients refractory to neomicyn treatment [177]. Furthermore, Malaguarnera [166] and Liu [172] confirmed these effects by using a combination of prebiotics and probiotics (a symbiotic approach) in the treatment of minimal hepatic encephalopathy. Bacterial translocation is also responsible for the increased portal pressure at the basis of hyperdynamic circulatory state and increased hepatic vascular resistance [178]. Probiotics can decrease blood portal pressure and bleeding risk [179]. These promising but not yet uniform results [180,181] were confirmed by Rincon et al., who after 6 weeks VSL#3 administration, reported reduced hepatic venous pressure gradient in liver cirrhosis patients [182].

The final and most dramatic stage of liver cirrhosis evolution can be hepatocellular carcinoma. There are a few promising studies on the role of probiotics in reducing the carcinogenetic process of hepatocellular carcinoma. An in vivo study reported that rats exposed to aflatoxin had a lower expression of *c-myc, bcl2, cyclin D1* and *rasp21* after *Lactobacillus rhamnosus GG* administration [183]. On the other hand, administration of a multistrain probiotic (namely, *Lactobacillus* and *Propionobacterium* species) did not change the urinary excretion of aflatoxin metabolite in healthy volunteers. These data suggest that probiotics administration might reduce the effects of aflatoxin and have a chemopreventive role in hepatocellular carcinoma [184]. However, further studies are required to clarify these limited data.

## 4. Conclusions

The increasing evidence of the role of gut microbiota in the development, maintenance and disruption of the immune system comes from animal and human studies. The liver, as a key organ in local and systemic immunity maintenance, is in strict contact with microbial antigens and gut microbiota derangement has a direct or indirect causative role on the development and progression of several liver diseases (Table 1). Thus, microbiota modulation consisting in the use of probiotics seems an appealing instrument for a safe immunity re-shaping in liver diseases.

Gut virome modulation on liver and systemic immunity for the treatment of viral- and immune-mediated hepatitis and hepatocellular carcinoma are more than promising. However, randomized controlled trials are needed to confirm animal and preliminary human studies. Understanding in depth the immunomodulatory role of the gut microbiota and virome in health and disease is also of prime importance to counteract pandemics such as that caused by the ongoing SARS-CoV-2 infection, as COVID-19-affected patients show not only respiratory distress syndrome but also multiorgan dysfunction including the liver.

## Figures and Tables

**Table 1:** Liver diseases and gut microbiota derangements.

Liver Disease	Gut Microbial Derangement
**ALD**	↓Butyrate-producing *Clostridiales* spp. ↓*Bacteroides* and *Lactobacillus* ↓*Lachnospiracea* and *Ruminococceae* ↑pro-inflammatory *Enterobacteriaceae* ↑*Fusobacteria*
**NAFLD/NASH**	↓*Prevotella* ↑*Firmicutes/Nacteroides* ratio ↑*Bacteroides* *and* *Ruminococcus* ↑*Escherichia coli,* *Bacteroides vulgatus* (namely, in liver cirrhosis stage)
**Autoimmune Hepatitis**	UC typical gut microbiota derangement (PSC) [68] ↑*E.coli rough form (PBC)*
**Liver cirrhosis**	↓*Bacteroidetes* and *Firmicutes* ↓*Lachnospiraceae,* *Ruminococceae* ↑*Enterobacteriaceae* ↑*Streptococcus* spp., *Veilonella* species ↑*Veilonella,* *Megasphera,* *Dialister,* *Atobium,* *Prevotella*
**HCC**	↓*Lactobacillus* spp. *Bifidobacterium* spp., *Enterococcus* spp. ↑*Escherichia coli* ↑*Clostridium*

Abbreviations: ALD: alcoholic liver disease; NAFLD: non-alcoholic liver disease; NASH: non-alcoholic steato-hepatitis; UC: ulcerative colitis; PSC: primary sclerosing cholangitis; PBC: primary biliry cholangitis; HCC: hepatocellular carcinoma; ↓: reduced; ↑: increased.
